# CaDENCE: a large-scale call disconnection event dataset from consumer Android devices

**DOI:** 10.1016/j.dib.2026.113255

**Published:** 2026-09-11

**Authors:** Pedro Matias, Rosiane de Freitas

**Affiliations:** Institute of Computing, Federal University of Amazonas, Av. Gen. Rodrigo Octávio, 6200 Setor Norte do Campus Universitário Coroado, Manaus AM, Brazil

**Keywords:** Android-based data collection, Dropped calls, Event-level dataset, Mobile network analytics, Voice service quality

## Abstract

This article describes CaDENCE, a large-scale curated dataset of call disconnection events derived from pre-existing device-level telemetry collected from consumer Android smartphones. The source records were generated by an event-driven software instrumentation system integrated into the device operating system and synchronized to a manufacturer-side cloud data warehouse before the research curation process. This instrumentation used Android telephony framework APIs and manufacturer-side monitoring components to collect quality-of-service information at call termination, including signal quality, radio access technology, network context, and service-state indicators. The released dataset was built by filtering, standardizing, pseudonymizing, and labeling these telemetry records to support dropped-call characterization and related mobile network and call performance analyses. CaDENCE comprises 125,532,358 event-level records collected over 98 days, from March 9 to June 14, 2024, covering devices running Android 13 and 14. The dataset focuses on call disconnection events associated with packet-switched voice services, including Voice over Long-Term Evolution (VoLTE), Voice over New Radio (VoNR), and Voice over Wi-Fi (VoWiFi). Each record contains temporal, device, software, mobile network, radio access technology, signal quality, and service-state attributes, along with the binary label is_drop, which distinguishes dropped-call events from non-drop call termination outcomes. The data are organized as Parquet files using a Hive-style date-partitioned layout under the data_by_date/ directory and are accompanied by metadata files that describe the schema, missingness, value ranges, and daily and network-level aggregates, as well as data processing scripts. Because the public release was produced using a daily capped export strategy that prioritized dropped-call records, its class distribution is enriched for dropped-call analysis and should not be interpreted as the original call-outcome distribution in the source telemetry. CaDENCE is suitable for reuse in studies on dropped-call characterization, radio access technology transitions, device-side network diagnostics, and machine learning methods applied to event-level mobile network data.

Specifications Table

**Table:** 

Subject	Computer Science
Specific subject area	Mobile Networks, Network Performance, Machine Learning, Telecommunications
Type of data	Table (.parquet) - filtered and processed; metadata files (.json, .csv)
Data collection	Call termination events were automatically recorded by software instrumentation integrated into the Android OS of consumer devices, using Android telephony framework APIs and manufacturer-side service-quality monitoring components. One consolidated event record was generated per call termination and synchronized daily to a cloud-based data repository. Records were curated through a five-stage SQL-based pipeline comprising extraction and tabularization, filtering and quality validation, derivation and standardization, enrichment and pseudonymization, and final labeling, sampling, and packaging.
Data source location	Institution: Federal University of Amazonas, Manaus-Amazonas Country: Brazil Latitude and longitude: 3°05′17.3″S 59°57′52.7″W
Data accessibility	Repository name: Zenodo Data identification number: 10.5281/zenodo.18918866 Direct URL to data: https://doi.org/10.5281/zenodo.18918866
Related research article	Matias, P. V. S., Miranda Filho, R., & de Freitas, R. (2024). Understanding the challenges of the call drop prediction problem in IP Multimedia Subsystem Networks. In Anais do XXI Encontro Nacional de Inteligência Artificial e Computacional (ENIAC) (pp. 263–274). SBC. https://doi.org/10.5753/eniac.2024.245284

## 1 Value of the Data

• The curated dataset contains >125 million event-level records of call disconnection outcomes collected from consumer Android devices under real-world operating conditions over a 98-day period and across multiple mobile network operators.
• Each record captures the call state at termination, including mobile network identifiers, radio access technologies across the call lifecycle, channel and band information, signal-quality measurements, and service-state indicators. These attributes support descriptive analyses of conditions associated with call disconnection outcomes in IP Multimedia Subsystem (IMS)-based voice services such as VoLTE, VoNR, and VoWiFi.
• The dataset includes records from devices running different hardware configurations and software versions. Pseudonymous product and build identifiers allow aggregated analyses across device and software contexts without exposing proprietary or user-level information.
• Organized at the call-event level rather than as continuous signal traces, the data can be reused by telecommunications practitioners and researchers for tasks such as dropped-call characterization, binary dropped-call classification, inter-RAT transition analysis, MCC/MNC-level descriptive aggregation, temporal analysis of call termination outcomes, failure-pattern exploration, and rare-event machine-learning experiments.
• The public release was produced using a daily capped export strategy that prioritized dropped-call records. Because the released class distribution is enriched for dropped-call analysis, the data should not be used to estimate population-level dropped-call rates, infer national network quality, perform user-level mobility analysis, or support causal claims about operators, devices, or firmware.

## 2 Background

A dropped call occurs when an ongoing voice call is interrupted before its normal completion. This event is related to quality-of-service (QoS) and quality-of-experience (QoE) problems in mobile voice networks [1,2]. In current packet-switched voice services, IMS provides the service architecture for voice over LTE (4 G), NR (5 G), and Wi-Fi calling [2]. Previous studies on call-drop, call-failure, and call-quality prediction have mainly focused on model development using IMS traces, LTE session records, or service and network parameters collected in specific contexts [[1], [2], [3]]. Although these studies show the relevance of the problem, their datasets are often not released as reusable event-level resources, which limits reproducibility and direct comparison across methods. Related data articles have made Android-based mobile network datasets publicly available for coverage and signal-quality analysis across devices, operators, and locations [4]. However, these datasets are broader network-measurement resources and are not centered on call termination outcomes. Unlike the related prior study by the authors [1], which focused on call-drop prediction and model evaluation, CaDENCE was compiled to address this gap by documenting a reusable device-side data resource organized around call disconnection events, with drop and non-drop outcomes, signal-quality measurements, and network context.

## 3 Data Description

The released repository is organized into four main components: event-level data, metadata, a preview sample, and supporting scripts. Event-level data are stored in data_by_date/ as *Parquet* partitions organized by date using a Hive-style layout (date=YYYY-MM-DD/), while auxiliary .csv files are stored in metadata/ and the curation logic files and supporting scripts are in scripts/. The complete Zenodo release is distributed as a single compressed archive of approximately 510 MB [7]. The organization of the repository and the role of each released file are summarized in Table 1.

**Table 1:** Repository contents and file descriptions.

Path	Description
data_by_date/date=YYYY-MM-DD/*.parquet	Event-level Parquet partitions organized by date (Hive layout).
sample/ cadence_preview_sample.csv	Preview CSV sample containing 9624 records selected from the released dataset to allow quick inspection.
metadata/schema.json	Column names and data types of the released table.
metadata/missingness.csv	Missing-value fraction for each column.
metadata/value_ranges.csv	Global minimum, selected quantiles, and maximum for selected numeric fields.
metadata/summary_by_day.csv	Daily aggregates computed over the released dataset.
metadata/summary_by_mcc.csv	Aggregates by MCC computed over the released dataset.
metadata/calldrop_warehouse_vs_released.csv	Daily comparison between warehouse and released Parquet counts to document the dropped-call capped export strategy.
scripts/metadata_generation/	Python scripts for loading the released Parquet files and reproducing descriptive metadata and summary statistics.
scripts/curation_etl_logic/	Anonymized pseudocode documenting the public-release ETL, curation, and export logic.
README.md	Dataset documentation, repository overview, and loading examples.
CITATION.cff	Citation metadata for the released dataset.
DATA_LICENSE.txt	License terms for dataset use and redistribution.

The metadata/ directory contains files that support navigation and inspection of the release. Specifically, schema.json lists column names and data types; missingness.csv reports the fraction of missing values per column; value_ranges.csv provides global summaries for selected numeric fields; and summary_by_day.csv and summary_by_mcc.csv provide aggregates by date and by MCC, respectively, computed over the released dataset. The file calldrop_warehouse_vs_released.csv compares daily warehouse and released counts to document the effect of the daily capped export strategy. Its released_* values are reproducible from the public Parquet files, whereas the warehouse_* values are author-provided aggregates from the non-public manufacturer-side warehouse. Additionally, the scripts/metadata_generation/ directory contains Python scripts that reproduce the release-derived metadata files from the released Parquet partitions, while the scripts/curation_etl_logic/ directory documents the public-release curation logic, data-collection assumptions, and outcome-consistency rules using anonymized pseudocode.

In the released table, all columns have 0% missing values after curation. This 0% missingness refers only to the public release and should not be interpreted as the natural completeness of the original telemetry source, because records with null values in core telemetry fields, including attributes not retained in the public release, were removed during the curation process. The value-range metadata include summaries for radio and signal fields, including RSRP, RSRQ, channel, and band; for example, RSRP ranges from −140 to −44 dBm and RSRQ ranges from −19 to −3 dB after plausibility filtering based on 3GPP-aligned radio-measurement ranges, including LTE RSRP/RSRQ reporting ranges from TS 36.133 [6]. Each row in the event-level table corresponds to one call disconnection event and contains attributes describing the network context, radio signal parameters, and service states at the time of call termination. The variables included in the released event-level table are described in Table 2.

**Table 2:** Data dictionary of the released event-level table.

Column	Type	Description
date	Date	Event date (daily granularity).
hour_of_day	Integer	Hour of day (0–23).
day_of_week	Integer	Day of week (1 = Sunday, 7 = Saturday).
android_version	Integer	Major Android version (13 or 14).
product_id	String	Persistent pseudonymous product identifier used in the released dataset; no reverse mapping to commercial product names is provided.
build_id	String	Persistent pseudonymous software/build identifier used in the released dataset; no reverse mapping to internal or commercial build names is provided.
mcc	String	Mobile Country Code.
mnc	String	Mobile Network Code.
initialRAT	Integer	Android Telephony API radio access technology code observed at call setup or call reception [5].
activeRAT	Integer	Android Telephony API radio access technology code observed while the call was active [5].
disconnectRAT	Integer	Android Telephony API radio access technology code observed at call termination/disconnection [5].
rat_handover	Boolean	Derived boolean indicating whether the RAT code differed among initialRAT, activeRAT and disconnectRAT
band	Integer	Radio Frequency Band of camped/active cell.
channel	Integer	Radio channel number of the active cell.
rsrp	Integer	Reference Signal Received Power (RSRP), reported in dBm (3GPP TS 36.133 [6]).
rsrq	Integer	Reference Signal Received Quality (RSRQ), reported in dB (3GPP TS 36.133 [6]).
wifi_st	Boolean	Wi-Fi interface connected at the time of the event; it does not necessarily imply that the voice bearer was routed over VoWiFi.
ims_reg	Boolean	Boolean indicating the IMS registration state reported at or near call termination.
roam	Boolean	Flag indicating if the device was operating on a roaming network during the call.
is_drop	Boolean	Binary dropped-call outcome label derived from curated call outcome and termination-context rules.

The RAT variables describe radio access technology at three observed stages of the call lifecycle: initialRAT, activeRAT, and disconnectRAT. The derived field rat_handover indicates whether the RAT code differed between these observed stages. Because RAT is sampled only at these stages, this field captures inter-stage RAT changes but does not represent continuous intra-call handover dynamics and does not specifically identify CS fallback, EPS fallback, or other protocol-specific transition mechanisms. Table 3 summarizes the Android Telephony API RAT codes observed in each released RAT field.

**Table 3:** Observed android telephony API RAT codes in the curated dataset.

Code	RAT meaning	initialRAT	activeRAT	disconnectRAT
0	UNKNOWN	50,954,614 (40.59%)	823,306 (0.66%)	—
3	UMTS	18,160 (0.01%)	112 (∼0.00%)	—
4	CDMA	1 (∼0.00%)	—	—
7	1xRTT	2 (∼0.00%)	—	—
13	LTE	57,212,438 (45.58%)	106,732,455 (85.02%)	110,085,287 (87.69%)
16	GSM	10,260 (0.01%)	91 (∼0.00%)	—
18	IWLAN / Wi-Fi calling	88,844 (0.07%)	11,304,510 (9.01%)	8,285,329 (6.60%)
20	NR	17,248,039 (13.74%)	6,671,884 (5.31%)	7,161,742 (5.71%)

Table 4 summarizes the main composition statistics of the released dataset. These statistics are provided to support reuse and to present the class distribution, temporal coverage, Android versions, and network and device diversity represented in the public release.

**Table 4:** Dataset composition summary.

Statistic	Value
Total event-level records	125,532,358
Dropped-call records (is_drop = True)	21,901,334 (17.45%)
Non-drop records (is_drop = False)	103,631,024 (82.55%)
Collection period	2024–03–09 to 2024–06–14 (98 days)
Released variables	20
Android versions available	13/T (Tiramisu), 14/U (Upside Down Cake)
Distinct product_id values	43
Distinct build_id values	692
Distinct Mobile Country Codes (MCCs)	159
MCC/MNC pairs	555

The percentages reported for dropped-call and non-drop records refer to the released dataset and should be interpreted together with the sampling procedure described in Section 4.2.5. They should not be interpreted as the original call-outcome distribution in the source telemetry. MCC values provide geographic context, and MCC/MNC pairs provide PLMN-level network context. Specifically, the low-count pairs were not suppressed solely because of their frequency, and they were retained when the corresponding records passed the general curation filters. These fields should not be interpreted as exact counts of countries or mobile service providers because some countries or territories may be associated with multiple MCCs, and some virtual operators may share PLMN identifiers used by host carriers.

The released dataset spans 159 distinct MCCs, but the record distribution is concentrated: the three most frequent MCCs account for approximately 70% of all records, and the ten most frequent MCCs account for approximately 91%. This concentration should be considered when interpreting geographic or PLMN-level aggregates.

As a practical example, one record included in the preview file sample/cadence_preview_sample.csv represents a call termination event recorded on May 15, 2024, between 13:00 and 13:59, on a device represented by product_id P0037 and build_id B00205, connected to MCC 310 (United States) and MNC 260, associated with T-Mobile US and also used by MVNOs operating on its network. The record reports initialRAT = 20 (NR), activeRAT = 13 (LTE), and disconnectRAT = 13 (LTE), indicating that the RAT differed between the observed call stages (rat_handover = True). At or near termination, the reported measurements were rsrp = −92 dBm and rsrq = −9 dB, with wifi_st = False, ims_reg = True, and roam = False. The event was assigned is_drop = True, indicating a dropped-call outcome. Together, these fields illustrate how a single row combines temporal, device, software, network, radio, signal-quality, and service-state information with the curated call outcome. The preview file contains 9624 released records and is provided for schema inspection and loading tests rather than statistical analysis.

## 4 Experimental Design, Materials and Methods

**Fig. 1:**
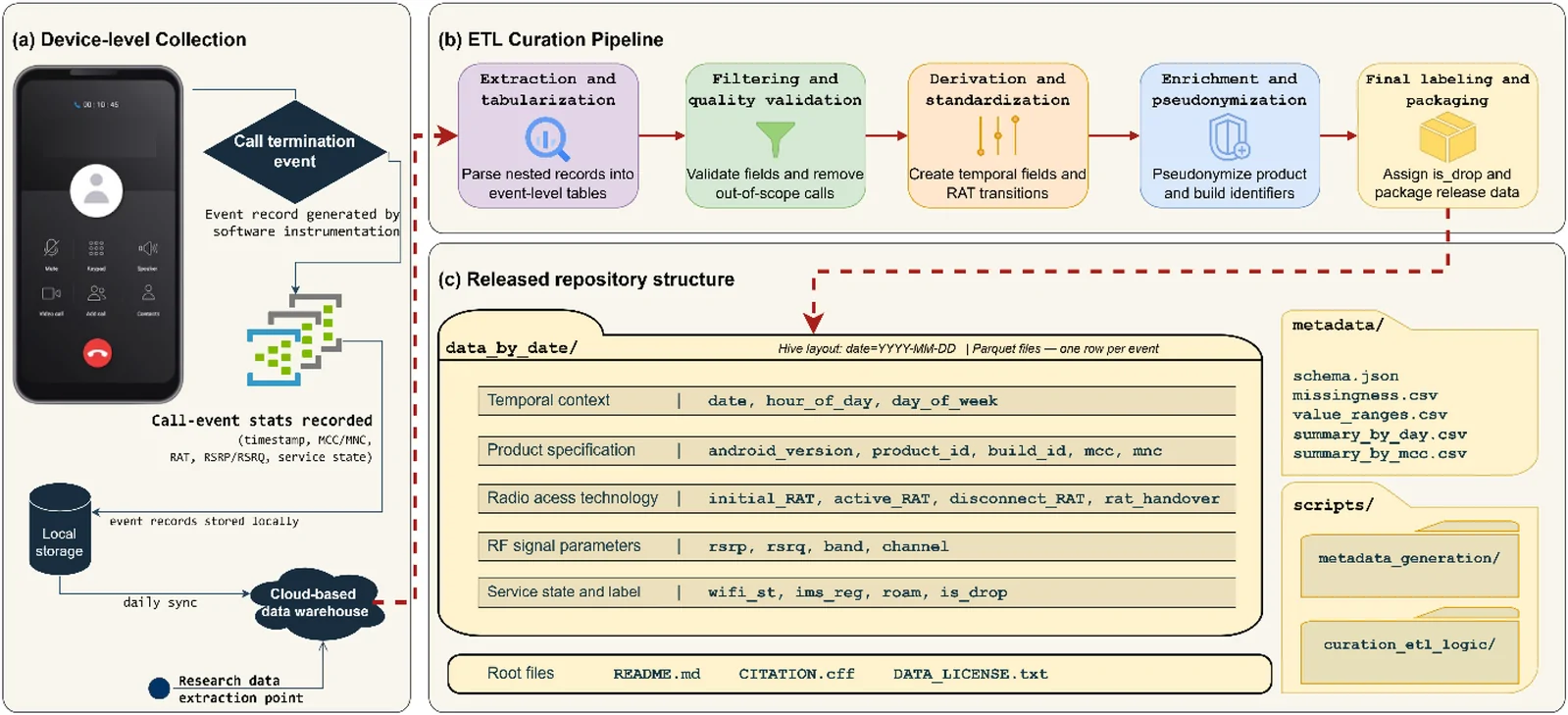
Overview of the device-level collection, curation, and release workflow. Panel (a) shows event-driven call-termination records stored locally and synchronized daily to a cloud-based warehouse. Panel (b) summarizes the ETL curation pipeline. Panel (c) presents the released repository structure.

This section describes the workflow adopted to build the released event-level dataset of call disconnection events from device-level records collected through software instrumentation. As summarized in Fig. 1, the workflow is organized into three components: device-level collection, in which a consolidated event record is generated at call termination; an Extract, Transform, and Load (ETL) curation pipeline, comprising extraction and tabularization, filtering and quality validation, derivation and standardization, enrichment and pseudonymization, and final labeling and packaging; and the released repository structure. The description below focuses on the attributes retained in the final public release. Additional diagnostic fields present in the raw records were used only as internal support during curation and were not included in the released dataset.

### 4.1 Device-level collection

The data were obtained from event-driven software instrumentation integrated into the Android operating system. Leveraging the telephony framework and internal APIs, this proprietary component passively generated a single consolidated record at the end of each voice call. Information recorded during the setup and active phases was limited to the serving carrier and the radio access technology in use, while radio measurements and service-state indicators were captured as a snapshot at the time of disconnection.

These records were stored locally on the device and synchronized daily with the manufacturer's cloud-based data warehouse. The present work relied exclusively on this existing warehouse; the authors did not modify or have access to the underlying proprietary instrumentation (e.g., specific intents or callbacks used to detect state transitions). All analyzed records originated from devices where diagnostic data sharing was enabled by the user during initial setup. To ensure privacy, no personally identifiable information was included in the public release, and proprietary product or software identifiers were pseudonymized.

### 4.2 Extract, transform, and load (ETL) curation pipeline

The released event-level table was constructed from previously collected device-level records stored in the data warehouse. As summarized in Fig. 1, the curation workflow begins with data extraction, filtering, and quality validation, then proceeds to attribute standardization, pseudonymization, and final packaging.

#### 4.2.1 Extraction and tabularization

Raw event records were stored in daily cloud tables as nested event structures and converted into a tabular event-level representation. The extracted attributes included the event timestamp, mobile network identifiers, radio access technology codes observed during the initial, active, and disconnection stages of the call, frequency channel and band, signal-quality measurements, and device connectivity and service-state indicators.

Only records from devices reporting instrumentation version 3.0 or higher were retained. Records missing values for radio access technology, rsrp, rsrq, channel, or band were excluded from further processing. A total of 4,433,398,934 raw records were extracted and tabularized for subsequent curation steps.

#### 4.2.2 Filtering and quality validation

At this stage, out-of-scope and technically inconsistent records were removed. Records corresponding to video calls, calls that never reached an active state, and observations with missing values in core network and signal fields were excluded. Only events whose disconnection stage was associated with LTE, NR, or Wi-Fi calling were retained.

Signal-quality measurements were screened using 3GPP-aligned plausibility ranges based on LTE RSRP/RSRQ reporting ranges from TS 36.133 [6]. The applied thresholds were RSSI between −113 and −51 dBm, RSRP between −156 and −44 dBm, and RSRQ between −19.5 and −2.5 dB. Of these fields, only rsrp and rsrq were retained in the final release.

Records whose source outcome category was inconsistent with the termination context reported by auxiliary diagnostic attributes were reclassified using deterministic consistency rules. These rules could change a non-drop outcome to a dropped-call outcome or the reverse. The auxiliary attributes were used only during curation and were not retained in the public release.

The anonymized curation pseudocode available in scripts/curation_etl_logic/ documents the consistency rules without exposing proprietary source tables or internal identifiers. Table 5 summarizes the main filtering and curation criteria.

**Table 5:** Main filtering and curation criteria applied before the public release.

Criterion	Action
Software instrumentation version on the device lower than 3.0	Excluded
Missing values in core release fields, including RAT, RSRP, RSRQ, channel, or band	Excluded
Video call event	Excluded
Call event that did not reach an active state	Excluded
Disconnection RAT outside LTE, NR, or Wi-Fi calling contexts	Excluded
Signal-quality values outside of 3GPP ranges	Excluded
Inconsistent outcome category relative to termination context	Reclassified according to curation rules

After filtering and quality validation, 2,940,996,083 records remained. Label reconciliation changed the assigned outcome of 915,499 records, corresponding to approximately 0.031% of the records evaluated at this stage. No records were removed solely because of an unresolved outcome inconsistency. Table 6 summarizes these transitions.

**Table 6:** Outcome-label transitions during label reconciliation.

Label transition	Records	Percentage of records evaluated
Non-drop to drop	910,769	0.03097%
Drop to non-drop	4,730	0.00016%
**Total reclassified**	**915,499**	**0.03113%**

#### 4.2.3 Derivation and standardization

Once filtering was complete, the remaining records were transformed to match the structure of the released event-level table. Temporal variables were derived from the original event timestamp, and the major Android version was inferred from the build identifier. Radio access technology attributes were standardized across the three observed call stages, and a binary indicator was derived to identify calls in which the radio access technology changed between stages. Signal quality and service-state variables were standardized to a consistent representation across instrumentation versions. After the transformations and scope checks associated with this stage, 2,834,328,932 records remained.

#### 4.2.4 Enrichment and pseudonymization

The curated records were then prepared for public release. Internal product and software identifiers were replaced with the pseudonymous fields *product_id* and *build_id*, while mobile network identifiers were retained in the released table as mcc and mnc. The pseudonym assignment was applied uniformly across all partitions, so each identifier maps to the same pseudonym throughout the collection period, and no reverse mapping table was distributed with the released files.

#### 4.2.5 Final labeling and packaging

The binary target variable is_drop was derived from the final event classification, taking the value True for dropped-call events and False for the remaining non-drop outcomes. The source records contained multiple outcome categories, including dropped calls, successful calls, and call setup failures. The outcome-consistency rules applied before final labeling are described in Section 4.2.2. Records missing core telemetry fields required by the curation pipeline were removed before packaging.

The final dataset was packaged in Parquet format, with one row per event and date-based partitioning using a Hive-style layout (date=YYYY-MM-DD/). Due to export constraints, each daily extract was limited to up to 2 million records. Within each daily partition, dropped-call records were selected first, and the remaining daily quota was filled with eligible non-drop records without a secondary ordering criterion or random seed. Therefore, the retained non-drop subset should not be interpreted as a random sample, as the specific records included may depend on the ordering of the source query results among eligible non-drop records.

Table 7 summarizes record retention across the curation pipeline, while Table 8 compares warehouse and released-dataset counts. The curation-stage counts in Table 7 and the warehouse counts in Table 8 originate from the non-public manufacturer-side warehouse, whereas the counts for the released dataset can be independently verified from the public Parquet files.

**Table 7:** Records retained after each stage of the curation pipeline.

Curation stage	Section	Records
Records extracted and tabularized	4.2.1	4,433,398,934
After filtering, quality validation, and label reconciliation	4.2.2	2,940,996,083
After derivation, standardization, and scope checks	4.2.3	2,834,328,932
After enrichment and pseudonymization	4.2.4	2,834,318,577
Final curated population	4.2.5	2,834,312,923
Released dataset after capped daily export	4.2.5	125,532,358

**Table 8:** Comparison between the manufacturer-side warehouse and the released dataset.

	Warehouse (data source)	Released dataset	Retained
Dropped-call records	22,091,188 (0.50%)	21,901,334 (17.45%)	99.14%
Non-drop records	4,411,307,746 (99.50%)	103,631,024 (82.55%)	2.35%
**Total records**	**4,433,398,934**	**125,532,358**	**2.83%**

As shown in Table 8, the release retained nearly all dropped-call records but only a small fraction of non-drop records. Consequently, the released class distribution is enriched for dropped-call analysis and should not be interpreted as the original call-outcome distribution in the source telemetry.

### 4.3 Released repository structure

The released repository includes event-level data files, metadata files, a preview sample, supporting scripts, and root-level documentation files. Event-level data are stored under ***data_by_date/*** using a Hive-style partitioning layout (*date=YYYY-MM-DD/*), with one Parquet file per partition and one row per event. The released table contains 20 variables covering temporal context, device and network identifiers, radio access technology attributes, signal quality metrics, service-state indicators, and the binary target label is_drop. The full variable descriptions are provided in Table 2.

The repository also includes a metadata/ directory containing auxiliary files to support dataset interpretation and reuse: schema.json, missingness.csv, value_ranges.csv, summary_by_day.csv, summary_by_mcc.csv, and calldrop_warehouse_vs_released.csv. The last file provides aggregate warehouse-versus-release counts used to document the effect of the daily export strategy described in Section 4.2.5. Supporting scripts are provided under scripts/, including scripts for metadata generation and anonymized curation-logic documentation. Root-level documentation files include *README.md, CITATION.cff*, and *DATA_LICENSE.txt*.

## Limitations

The released dataset is derived from device-level records collected through proprietary software instrumentation integrated into consumer Android devices (whose internal implementation was not accessible to the authors). Although the data cover different device models, hardware configurations, software builds, markets, and radio technologies, all records originate from devices of a single manufacturer. Therefore, the dataset should not be interpreted as representative of all Android devices, manufacturers, regions, or mobile network conditions.

The geographic and network coverage of the release should also be interpreted with caution. MCC values and MCC/MNC pairs provide useful geographic and PLMN-level context, but the distribution of records is not geographically uniform and reflects the records available in the source telemetry after the scope and curation criteria applied in this work. Consequently, MCC/MNC aggregates should not be used to infer national network quality, operator-level performance, or population-level service conditions.

The public release was restricted to curated event records with sufficient completeness in the core fields, which may exclude calls with partial telemetry. In addition, the sampling procedure described in Section 4.2.5 affects the class distribution of the released dataset. Analyses based on the release should therefore consider that the proportions of dropped-call and non-drop records reflect the released sample and not the original distribution of call outcomes in the source telemetry.

Several variables should be interpreted as event-level observations rather than continuous traces. The RAT fields capture radio access technology at observed stages of the call lifecycle, and the rat_handover field only indicates differences between these observed states. It should not be interpreted as a continuous intra-call handover trace or as evidence of a specific fallback mechanism, such as CS fallback or EPS fallback. Similarly, the dataset does not include explicit mobility state, vehicular context, or continuous location traces.

The pseudonymous product_id and build_id fields support aggregated analyses across device and software contexts, but no reverse mapping to commercial product names, internal build names, modem/baseband firmware versions, or 3GPP Release capabilities is provided. Although consistency rules were applied during curation, no independent event-level ground truth was available. The approximately 0.031% reported in Section 4.2.2 quantifies records reclassified by these rules rather than absolute label accuracy. Residual label noise may therefore remain, and its magnitude cannot be quantified from the available data.

## Ethics Statement

The dataset described in this article is based on technical device-level records collected through software instrumentation from consumer devices for which diagnostic data sharing had been enabled by the user during device setup. The authors did not conduct experiments with users, did not interact with users, and did not collect new data directly from human participants. The study used previously collected technical telemetry made available within a formal university–industry research collaboration.

Access to the original telemetry was restricted to the data owner’s controlled environment. The public dataset was produced through a curation process designed to release only a pseudonymized and reduced event-level version of the original records. The released data do not contain personally identifiable information or sensitive personal data. Fields excluded from the public dataset include device-level identifiers and internal diagnostic fields developed by the manufacturer and used only during curation. Proprietary product and software identifiers were replaced by pseudonymous fields before publication, and no reverse mapping table was distributed with the released files.

Because the work consisted of secondary analysis and publication of curated, pseudonymized technical telemetry, and did not involve human-subject experiments, surveys, interventions, or the release of personal or sensitive information, no additional formal ethics review was required. The study did not involve experiments with animals.

## Credit Author Statement

**Pedro Matias**: Conceptualization, Methodology, Data curation, Investigation, Validation, Visualization, Writing – original draft; **Rosiane de Freitas**: Project administration, Supervision, Writing – Reviewing and Editing.

## Data Availability

ZenodoCaDENCE: a large-scale call disconnection event dataset from consumer Android devices (Mar-Jun 2024) (Original data)
